# Outcomes of Surgical Exploration of Acute Scrotal Pain Raising Suspicion of Testicular Torsion

**DOI:** 10.7759/cureus.40098

**Published:** 2023-06-07

**Authors:** Sayed Hasan Ebrahim, Ali A Alaysreen, Yousif F Yousif

**Affiliations:** 1 Urology, Salmaniya Medical Complex, Manama, BHR

**Keywords:** orchidopexy, orchiectomy, scrotal exploration, doppler ultrasound, testicular torsion

## Abstract

Background and objective

Testicular torsion (TT) is a surgical emergency, affecting approximately one in every 4000 males under the age of 25 years annually in the United States. In this study, we aimed to determine the outcomes of emergency scrotal surgical exploration of cases that raised suspicion of TT presenting to Salmaniya Medical Complex (SMC), Bahrain's largest secondary and tertiary care center.

Methods

This was a retrospective cohort study. The data were collected from the hospital's electronic medical record software (I-SEHA). The data included patient age, Doppler ultrasound (DUS) findings prior to any surgical procedure, type of surgical procedure, and the surgical findings of that procedure.

Results

Of the 198 patients who underwent scrotal exploration, 141 had presented with signs and symptoms suggestive of TT. The mean age of the patients was 22.3 ±9.3 years. Doppler imaging was performed preoperatively on 135 out of 141 (95.7%) patients. After scrotal exploration, 91.4% of the patients were found to have TT. The proportion of patients with salvageable testis was 78.7%.

Conclusions

The study found that surgical exploration remains the definitive method in the management of acute scrotum in TT patients. Our findings are in line with those from other similar studies and meta-analyses.

## Introduction

Testicular torsion (TT) is a surgical emergency, and it affects approximately one in every 4000 males under the age of 25 annually in the United States [1]. Patients usually present to the emergency department with acute severe testicular pain that is mostly unilateral and associated with nausea, vomiting, and lower abdominal pain [2]. On physical examination, loss of cremasteric reflex is suggestive of TT, with 100% sensitivity and 66% specificity for the condition [3]. Other findings include high-riding testicles, which indicate a twisted foreshortened spermatic cord [4].

The diagnosis of TT is mainly based on clinical suspicion; however, other diagnostic modalities such as Doppler Ultrasound (DUS) can also be used to assist in the diagnosis. DUS findings such as decreased or absent blood flow are suggestive of TT [5]. However, some DUS findings may also be misleading. A multicenter study has shown that 24% of the patients with acute TT had normal or increased testicular vascularization [6].

Patients with TT require urgent surgical intervention to restore blood flow to the ischemic tissue. The duration between the onset of symptoms and detorsion, as well as the degree of cord twisting, are the two most important determinants of the early salvage rate of the testis [7]. There is typically a four-to-eight-hour window before significant ischemic damage occurs [8]. However, this window could be shorter depending on the degree of cord twist, and severe testicular atrophy could occur within as early as four hours for torsions with turns >360 degrees [9].

According to the European Association of Urology (EAU), manual detorsion of the testis should be performed initially, as it is associated with higher surgical salvage rates [10,11]. However, the definitive treatment of TT is scrotal exploration. Urgent scrotal exploration is mandatory for all patients within 24 hours of symptom onset; however, after 24 hours, it may be performed as a semi-elective exploration procedure [11]. During surgical exploration, detorsion of the affected spermatic cord and orchiopexy is done if the testis appears viable, with orchiectomy being performed if the affected testis appears grossly necrotic or non-viable. Orchiopexy is also performed on the contralateral testis.

In this study, our objective was to find the outcomes of emergency scrotal exploration in patients who presented with acute scrotal pain that raised suspicion of TT at Salmaniya Medical Complex (SMC), Bahrain's largest secondary and tertiary care center. We also assessed the efficacy of the preoperative utilization of DUS to aid in the diagnosis.

## Materials and methods

This retrospective cohort study was conducted at SMC Bahrain. Ethical approval was obtained from the Research and Research Ethics Committee for Government Hospitals on May 9, 2023. Data were collected using the hospital's electronic medical record software (I-SEHA). The sample initially included 223 patients booked in the system for bilateral scrotal exploration by the Urology Department between November 2018 and April 2023. All duplicate bookings and all patients who had undergone scrotal exploration for reasons other than TT, such as epididymo-orchitis, testicular abscess, scrotal trauma, and Fournier's gangrene, were excluded from the study. Details of the above-mentioned excluded patients are presented in Table 1. In addition, all patients who were under the age of 14 years were excluded from the study as those patients had been referred to the pediatric surgery department at our institute. Data from the remaining 141 patients were collected using I-SEHA. The data included patient age, if DUS was done before surgical exploration, the DUS findings that were documented in the system, the type of surgical procedure (orchidopexy or orchiectomy), and the documented surgical findings of that procedure, such as the degree of the rotation and the viability of the testis.

**Table 1:** Details of excluded patients

Total excluded	Reason for exclusion	Percentage (approximate)
25	Duplicated entries	30%
14	Epididymo-orchitis	17%
11	Scrotal wall abscess	13.4%
11	Trauma	13.4%
7	Fournier's gangrene	9%
4	Intra-testicular abscess	5%
4	Incorrect code of entry	5%
1	Testicular infarction	1.2%
1	Scrotal wall cellulitis	1.2%
1	No data found in the system	1.2%
1	Fistula	1.2%
1	Scrotal wall edema	1.2%
1	Refused surgery	1.2%

Following data collection, all data were analyzed using IBM SPSS Statistics for Mac, Version 27.0 (IBM Corp., Armonk, NY).

## Results

**Figure 1:**
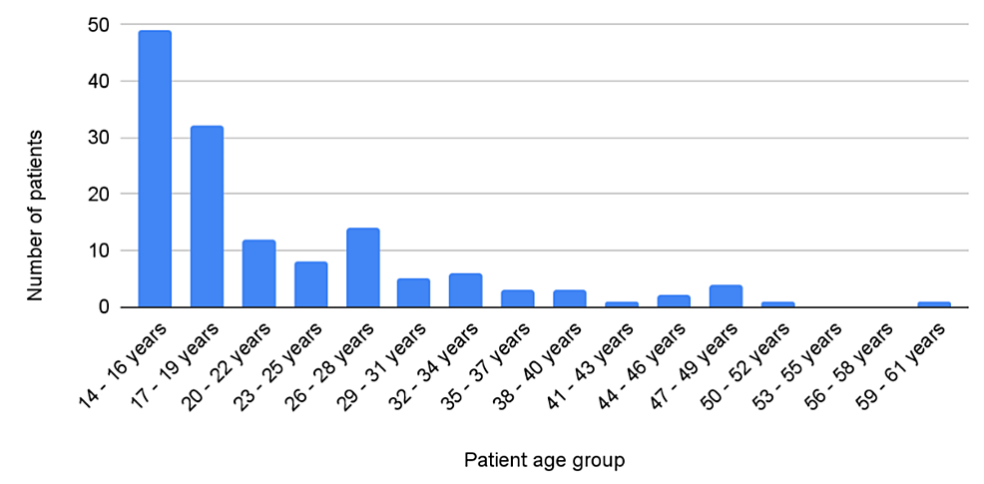
Number of patients who presented to the hospital with symptoms suggestive of testicular torsion by age group

Of the 198 patients who underwent scrotal exploration, 141 had presented with signs and symptoms suggestive of TT (Figure 1). The average age of patients was 22.3 ±9.3 years. The median age was 19, and the mode was 16. Figure 1 depicts the distribution of patients by age group.

DUS was performed preoperatively on 135 out of 141 (95.7%) patients who presented with signs and symptoms suggestive of TT. DUS findings were suggestive of TT in 132 (93%) patients. DUS sensitivity was 87.2%, while the specificity was 50%, the positive predictive value (PPV) was 95.6%, and the negative predictive value (NPV) was 31.25%. Table 2 presents the data on the rate of orchiectomy by age group.

**Table 2:** Rate of orchiectomy by patient age group

Patient age group	Number of patients	Number of orchiectomies
Less than 21 years	86	16 (18.6%)
21 years or more	55	14 (25.4%)

**Figure 2:**
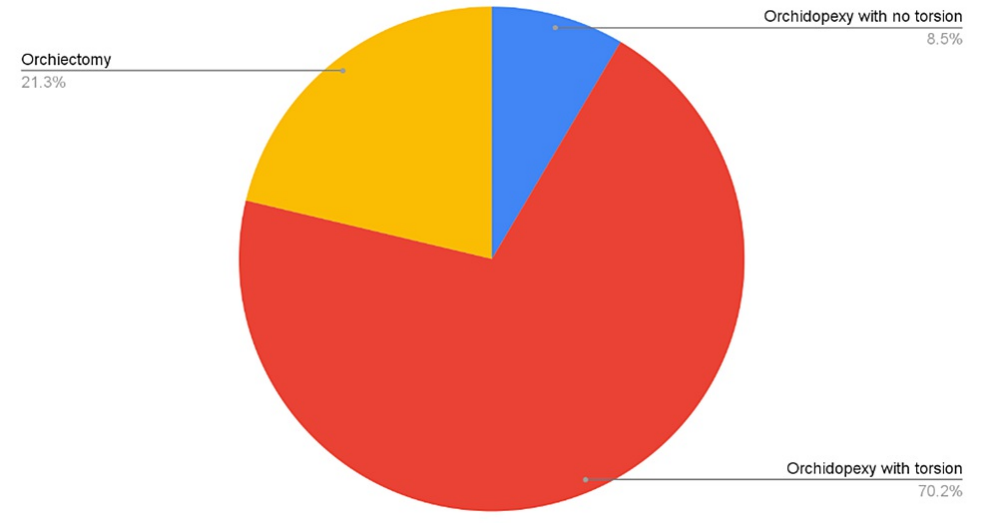
Outcomes of scrotal exploration

Of the 141 patients who underwent scrotal exploration due to the suspicion of TT, 12 (8.5%) were found not to have TT after surgical exploration (Figure 2). Of those 12 patients, 11 patients underwent a preoperative DUS, and of them, three patients did not have findings suggestive of torsion in their DUS.

Of the 141 patients who underwent scrotal exploration, 30 (21%) patients were found to have unsalvageable testis and underwent orchiectomy. The average age of those patients was 24.96 ±11.6 years. Out of those patients, 15 (50%) were found to have testis twisted at 360 degrees or more.

None of the patients who underwent orchidopexy needed further exploration in the subsequent follow-up at our institution and nor did they have any recurrence of torsion.

## Discussion

**Figure 3:**
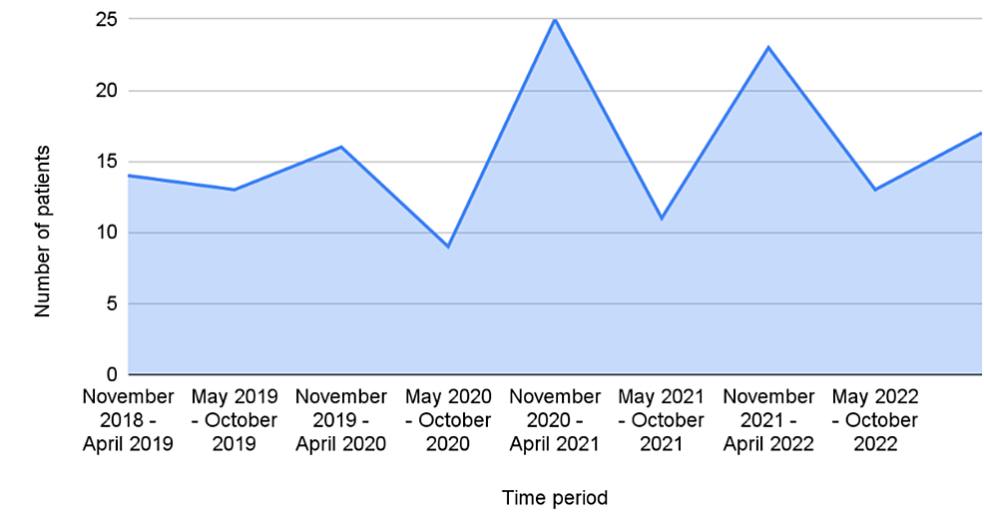
Number of patients by the time period of presentation

If we compare the number of cases based on the date of presentation, we notice that most patients (67.4%) presented between November and April compared to the period between May and October (Figure 3). These months tend to be colder in Bahrain; thus, we notice an increase in the incidence of TT during cold weather. Similar findings have been observed in a study in Brazil that included 21,289 hospital admissions for TT, which found a higher number of TT patients during colder months [12].

DUS is used as a diagnostic aid in patients presenting with acute scrotal pain, and its use is recommended by EAU [11]. However, although DUS could be helpful, it should not deter surgical exploration if there is a high clinical suspicion of TT [13]. This is evident in our study, as although 93% of the DUS performed revealed findings suggestive of TT when comparing the DUS findings to the surgical findings, we find that the test sensitivity was 87.2%, while the specificity was 50%, PPV was 95.6%, and NPV was 31.25%. This is comparable to a meta-analysis by Ota et al. on the role of ultrasound imaging in patients with TT, which included 2116 patients from 26 studies, revealing a specificity of 95% and sensitivity of 86% [14].

In our study, 12 patients who underwent scrotal exploration were found not to have TT. It is unclear if those patients had torsion-detorsion or presented with signs and symptoms mimicking TT. Looking at the surgical findings of those 12 patients, seven had hydrocele with or without varicocele, two had inguinal hernias, one had edematous testis, and two had normal testis. Hydrocele and varicocele can present with scrotal plain, thus mimicking symptoms of TT [15]. Torsion-detorsion can occur when there is obstruction of blood flow due to twisting of the testis, followed by blood-flow recovery as the testis returns to its normal position [16]. This phenomenon was observed in a case study where torsion-detorsion was found in a patient with acute scrotal pain with initial normal DUS findings that later resolved and returned hours later with DUS findings of decreased blood flow [17].

Our study had 30 patients who were found to have unsalvageable testis and underwent orchiectomy (Table 2). A reason for this could be the degree of twisting, as 50% of those patients had testis twisted at 360 degrees or more [18]. Filho et al. found that orchiectomy rates increased at 540 degrees, and testes were salvaged in all patients with less than 360 degrees of twist and removed in all patients with over 180 degrees of twisting [19]. Another study found that non-salvageable testicles tend to have a significantly higher degree of twisting when compared to salvageable testicles [20].

Our study had a salvage surgery rate of 78.7% for TT. This is higher than the salvage rate of 58.4% and 70.9% found in a multi-institutional study by Chun et al. [21]. This is also better than the 55.3% rate in a study by Ramachandra et al. conducted at a tertiary center [18]. In a systematic review by van Welie et al. on 46 patients from different case reports and series, the recurrence of TT was found to be a rarely reported event [22]. In our study, which covered a period of almost five years, no reported cases of TT recurrence were detected. The current practice in the hospital is using 3-point fixation involving non-absorbable sutures. Fixation of the tunica albuginea to the dartos muscle and eversion of the tunica albuginea has been shown to prevent retorsion, at least in the currently available literature [23].

The strengths of this study included the readily available Doppler scrotal ultrasound findings with 24-hour in-house coverage by senior radiologists that promptly ensured that all patients were being worked up without delay before surgical exploration. However, all cases with a high index of clinical suspicion of TT were surgically explored regardless of the findings of the scrotal DUS. Another advantage of this study is the clear documentation of the relevant aspects mentioned in this study in the medical electronic software system since 2018. Moreover, urologists and radiologists were involved early and simultaneously upon the presentation of acute scrotal pain cases with high clinical suspicion of TT.

This study has a few limitations. Primarily, the surgical notes were handwritten before 2018, and computer documentation using I-SEHA was optional. This made getting detailed notes of the surgical findings more difficult. There needs to be more emphasis on documentation of the duration of the presentation time to the time of surgical management. An audit comparing the duration of time from the patient's presentation to ER presentation, that from ER to the DUS, and then to the operation theater would be helpful in assessing the hospital's quality of care.

## Conclusions

This study sought to determine the outcomes of scrotal exploration in patients who presented to the Emergency Department of the SMC with acute scrotal pain that raised suspicion of TT. We also discussed the methods we adopt to manage these patients in our hospital setting. Our findings are comparable with those of other similar studies and meta-analyses. DUS that does not lead to a delay in surgical exploration is a useful adjunct to the management of these patients, and surgical exploration remains the definitive method to manage TT.
